# Association between albumin-bilirubin score and sarcopenia in middle-aged Americans: a cross-sectional study

**DOI:** 10.3389/fnut.2025.1606854

**Published:** 2025-07-04

**Authors:** Yifan Zhou, Jinghao Yu, Anping Wu, Haitao Tan, Jun Xiao

**Affiliations:** ^1^Department of Orthopedics, The 921st Hospital of the People’s Liberation Army, The Second Affiliated Hospital of Hunan Normal University, Changsha, China; ^2^Hangzhou Dianzi University, Hangzhou, Zhejiang, China; ^3^The No. 924 Hospital of the Joint Logistic Support Force of the Chinese People's Liberation Army, Guilin, China

**Keywords:** ALBI, sarcopenia, middle-aged adults, NHANES, liver function

## Abstract

**Background:**

The albumin-bilirubin (ALBI) score is a novel index for assessing liver function, integrating albumin and bilirubin levels to provide a comprehensive evaluation of a patient’s overall health. Despite its utility, there is a paucity of research exploring the relationship between ALBI scores and sarcopenia in the existing literature, and studies on sarcopenia prevalence in middle-aged adults are also scarce. This study aims to investigate the association between ALBI scores and the prevalence of sarcopenia among middle-aged adults in the United States.

**Methods:**

Utilizing data from the National Health and Nutrition Examination Survey (NHANES) collected from 2011 to 2018, participants were classified into prevalent and non-prevalent groups based on sarcopenia status. We constructed one univariate model and two multivariate models, employing restricted cubic spline analysis and weighted logistic regression to examine the relationship between ALBI scores and sarcopenia prevalence. Additionally, subgroup analyses assessed the association between ALBI scores and sarcopenia across various demographic groups. A random forest model was also developed to evaluate the importance of different variables related to sarcopenia, based on Gini impurity.

**Results:**

The study included a total of 10,354 participants. Analysis of demographic characteristics stratified by sarcopenia prevalence. In the fully adjusted model, a positive association was found between ALBI scores and sarcopenia prevalence [OR (95% CI): 4.28 (2.29, 8.01), *p* < 0.001]. Furthermore, subgroup analyses indicated no significant interactions across factors such as gender, race, education, marital status, diabetes mellitus, hypertension, smoking, and alcohol consumption. The random forest model revealed an important association between ALBI scores and sarcopenia.

**Conclusion:**

This study establishes a significant association between ALBI scores and sarcopenia prevalence in middle-aged adults in the United States. Our findings indicate that higher ALBI scores are correlated with an increased prevalence of sarcopenia, emphasizing the importance of incorporating ALBI scores into assessments of muscle health. These results suggest that ALBI scores could play a crucial role in informing preventive strategies and interventions to combat sarcopenia in this vulnerable population.

## 1 Introduction

Sarcopenia is defined as a progressive and systemic reduction in skeletal muscle mass and strength, is increasingly recognized as a syndrome that affects both physical performance and metabolic health (1–3). Although widely associated with older populations (4, 5), recent research shows that sarcopenia can begin in younger adults, particularly between middle-aged adults. Various studies indicate that up to 30% of middle-aged adults may exhibit signs of muscle degradation, often exacerbated by lifestyle factors such as sedentary behavior, poor nutrition, and chronic illnesses (6–9). As muscle mass declines, adults face increased risks of frailty, disability, and a higher likelihood of adverse health outcomes, including falls and hospitalizations (10). Moreover, sarcopenia is linked to metabolic disorders, such as insulin resistance and type 2 diabetes mellitus, heightening its impact on overall health and longevity (11, 12).

The liver serves as a vital metabolic organ, with its function closely associated with muscle mass and protein metabolism. Impaired liver function, particularly in cases of severe liver disease, can result in muscle loss and the development of sarcopenia (13, 14).

The evaluation of liver function has traditionally relied on a variety of biomarkers and scoring systems. Commonly used indicators include serum aminotransferases (ALT and AST), alkaline phosphatase, bilirubin levels, and prothrombin time (15–20). These parameters provide insight into liver inflammation, injury, and the capacity for synthetic function. However, while these traditional measures offer valuable information, they often fall short in providing a comprehensive evaluation of liver function, especially in cases of acute liver failure or cirrhosis. Recently, the ALBI (Albumin-Bilirubin) score has emerged as a novel tool for assessing liver function (21, 22). This scoring system is based on routine laboratory tests and combines serum albumin and total bilirubin levels to offer a straightforward and effective way to evaluate liver function and predict patient outcomes (23, 24). The ALBI score has been shown to correlate well with the traditional Child-Pugh score while requiring fewer laboratory parameters, making it particularly advantageous in clinical practice (25).

However, the correlation between ALBI score and prevalence of muscle loss is still not well understood.

In this study, we will explore the utility of ALBI score as a significant exposure factor in assessing outcomes related to the prevalence of sarcopenia. Providing evidence-based recommendations for this often-overlooked yet increasingly significant demographic is crucial. Gaining insight into these interactions can greatly influence public health strategies and programs aimed at preventing sarcopenia. This understanding facilitates the development of tailored lifestyle suggestions for middle-aged individuals who may be at risk of developing this condition.

## 2 Methods

### 2.1 Data source and population study

We utilized data from the National Health and Nutrition Examination Survey (NHANES), a cross-sectional, nationally representative study conducted by the Centers for Disease Control and Prevention (CDC). The NHANES employs a stratified, multistage, probabilistic cluster sampling design developed by the National Center for Health Statistics. Trained technicians conducted laboratory tests and various examinations, while interviewers administered questionnaires in participants’ homes. Our analysis encompassed data from four NHANES cycles from 2011 to 2018. Participants eligible for the study were required to be aged between 20 and 59 years and to have complete laboratory test results, which included albumin and bilirubin levels, as well as Dual-energy X-ray Absorptiometry (DXA) scans.

Ethical approval for NHANES was granted by the Institutional Review Board (IRB) of the CDC. All participants gave their informed consent before enrolling in the study, ensuring compliance with ethical standards throughout the data collection process.

**Figure 1:**
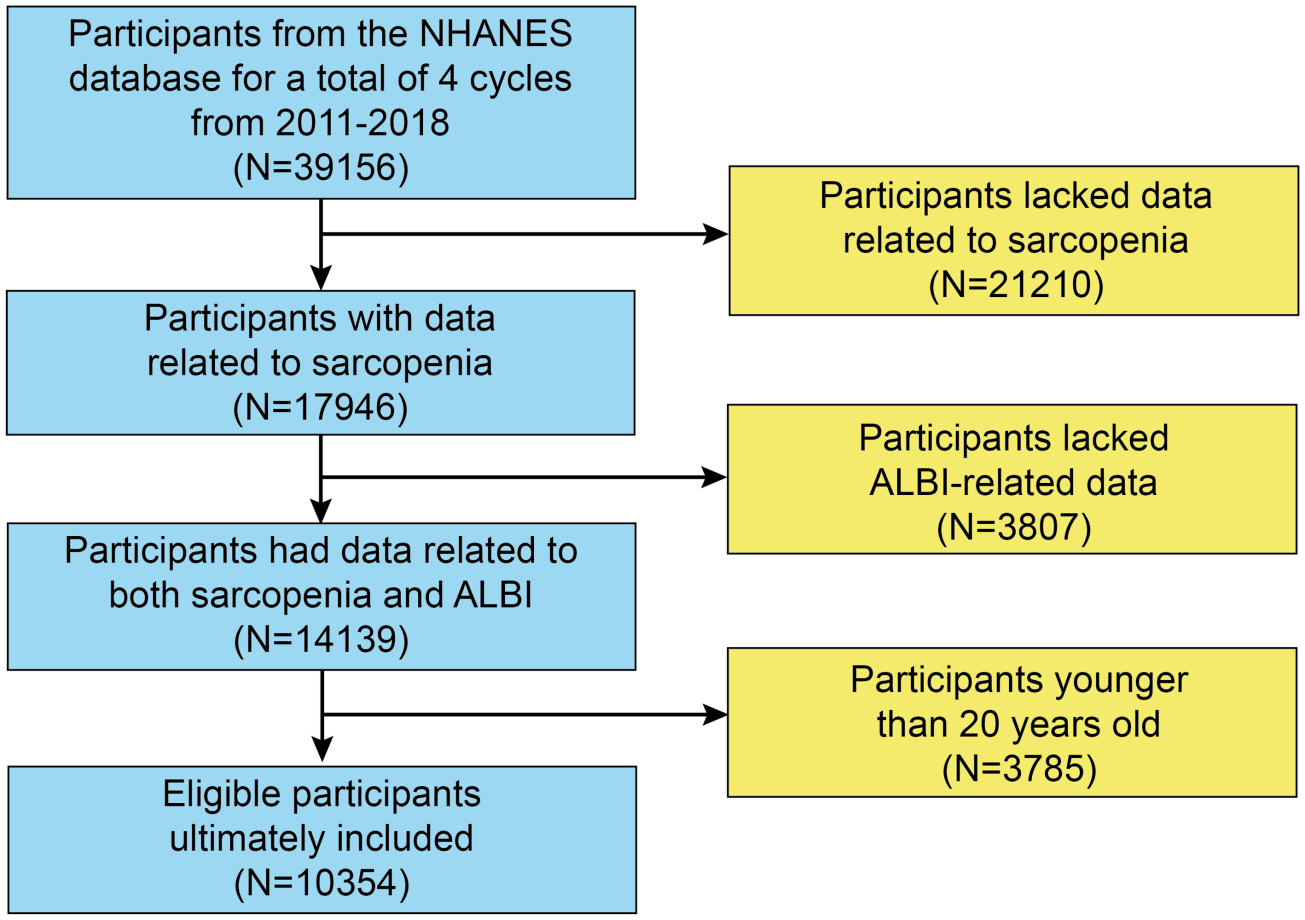
Flowchart of participants selection.

The initial sample consisted of 39,156 participants. However, 21,210 individuals did not undergo a DXA examination or body measurements, resulting in missing data for the Skeletal Muscle Mass Index (SMI). Therefore, 17,946 participants remained for further analysis. After excluding those without ALBI-related data (*n* = 3,807) and participants younger than 20 or older than 60 years (*n* = 3,785), our final analysis included 10,354 participants with complete datasets (Figure 1).

### 2.2 Measurement of exposure and outcome variables

#### 2.2.1 Definition of ALBI

The ALBI score is calculated using the following formula: ALBI = (log10 bilirubin × 0.66) + (albumin × − 0.085), where the total bilirubin is expressed in μmol/L and albumin in g/L.

According to officials from NHANES, the measurement of albumin concentration employs the dye bromcresol purple (BCP). This dye selectively binds to albumin within a pH range of 5.2 to 6.8, resulting in a detectable color change measured at a wavelength of 600 nm, with a secondary measurement at 700 nm. This method represents a two-point, endpoint reaction that specifically targets albumin.

For the determination of total bilirubin, the method involves coupling with 3,5-dichlorophenyl diazonium in a strongly acidic medium that contains a solubilizing agent. The intensity of the resulting red azo dye is directly proportional to the total bilirubin concentration, which can be quantified photometrically at a wavelength of 546 nm.

#### 2.2.2 Definition of sarcopenia

The diagnosis of sarcopenia generally consists of two main areas: muscle mass and muscle function. Foundation for the National Institutes of Health (FNIH) has published a set of diagnostic criteria for sarcopenia that are widely accepted and applied (4). This criteria emphasizes the measurement of muscle mass and function. The specific criteria are as follows: 1. Muscle mass: Muscle mass assessed using DXA (dual-energy X-ray absorptiometry). Skeletal Muscle Mass index (SMI): using the formula: SMI = Appendicular skeletal muscle mass (kg)/BMI (kg/m^2^) For the diagnosis of sarcopenia, the ASMI thresholds for men and women are, respectively, < 0.789 for men and < 0.512 for women. 2, Muscle Function: Grip Strength test: males: grip strength < 26 kg, females: grip strength < 16 kg.3, Gait speed test: a person may be considered to have sarcopenia if the walking time for 4 meters exceeds 6 s. In our study, we chose the more objective DXA examination of the muscle mass of the appendicular skeletal muscles to calculate the SMI value as the main diagnostic method for sarcopenia.

### 2.3 Covariates

Based on previous literature (26, 27), we included the following as covariates: demographics including age, gender, race, education (categorized as less than high school, high school, and more than high school), marital status (categorized as ever married and never married), and household poverty-to-income ratio (PIR), and other components including total protein, creatinine and smoking status (categorized by whether or not they have smoked 100 cigarettes in their lifetime), drinking status (by whether or not one has 4–5 drinks per day), hypertension and diabetes mellitus status (categorized by whether or not they have been diagnosed by a doctor with a related disease). Information on these covariates is available on the NHANES Web site (NHANES Questionnaires, Datasets, and Related Documentation (cdc.gov)). It is worth noting that because of the high correlation between ALT and AST with liver function and muscle mass, we did not include them as covariates in this study in order to avoid the occurrence of multicollinearity as much as possible.

### 2.4 Statistical analysis

Missing values for the remaining variables (e.g., creatinine, total protein, smoking status, drinking status) were filled in using multiple interpolation with the R package “missForest.”

Our analysis was conducted in accordance with the official NHANES guidelines, which include considerations for sample weights, clustering, and stratification. We initially examined the continuous variables for normality and determined that they were not normally distributed. Consequently, we presented continuous data as median values accompanied by interquartile ranges (IQR), whereas categorical variables were expressed as counts and percentages (%). Statistical analyses were performed using Mann–Whitney U tests for continuous variables and chi-square (χ^2^) tests for categorical variables. We established three models: the crude model, model 2 (which included demographic variables), and model 3 (which incorporated all relevant covariates). Weighted logistic regression models were employed to investigate the association between the Albumin-Bilirubin (ALBI) score and sarcopenia. Additionally, we investigated the possible nonlinear relationship through restricted cubic spline (RCS) analysis and performed a threshold effects analysis to pinpoint any inflection points. Additionally, subgroup analyses were carried out to evaluate the relationship between ALBI and sarcopenia across various populations, employing fully adjusted models for the stratified analysis of subgroup variables. Finally, to explore the importance of ALBI for sarcopenia, we built a random forest model and ranked the importance of different variables for sarcopenia according to the Gini impurity. All statistical analyses were conducted using R Studio (version 4.4.1)^1^, with a *p*-value of less than 0.05 deemed statistically significant.

## 3 Results

### 3.1 Characteristics of study population

Table 1 displays the demographic characteristics of participants stratified by their sarcopenia status. We analyzed data from the National Health and Nutrition Examination Survey (NHANES) collected between 2011 and 2018, ultimately including 10,354 participants in our study, which corresponds to an estimated population of 122,471,850 after applying inverse weighting. Our findings indicate that sarcopenia was more prevalent among Mexican Americans, individuals with lower educational attainment, and those diagnosed with hypertension or diabetes mellitus. Conversely, the prevalence of sarcopenia was significantly lower in Non-Hispanic Black individuals. Additionally, the BMI values of individuals with sarcopenia are significantly higher than those of individuals without the condition, with their BMI generally falling within the obesity range (BMI ≥ 30). Notably, people with sarcopenia have higher AST and ALT than those without sarcopenia, while their albumin and bilirubin levels are lower.

**Table 1:** Weighted characteristics of study populations in the NHANES (2011–2018) by sarcopenia status.

Characteristic	Overall *N* = 122,471,850	Non-sarcopenia *N* = 118,043,356	Sarcopenia *N* = 4,428,494	*P*-value
Age (year)	39.39 ± 11.68	39.23 ± 11.65	43.62 ± 11.64	<0.0001
Gender (%)				0.6118
Male	49.01	49.96	51.30	
Female	50.99	50.04	48.70	
Race (%)				<0.0001
Mexican American	15.11	9.91	28.95	
Other Hispanic	10.46	7.27	11.74	
Non-Hispanic White	34.71	61.93	45.19	
Non-Hispanic Black	20.91	11.41	3.59	
Other Race	18.81	9.48	10.53	
Education level (%)				<0.0001
Less than high school	6.23	3.50	14.42	
High school	11.9	8.83	13.10	
Above high school	81.87	87.67	72.48	
Marital status (%)				0.1556
Previously married	62.91	64.78	68.35	
Never married	37.09	35.22	31.65	
BMI (kg/m^2^)	28.77 ± 6.69	28.47 ± 6.44	36.76 ± 8.07	<0.0001
PIR	2.93 ± 1.63	2.96 ± 1.63	2.19 ± 1.43	<0.0001
Creatinine (mg/dl)	0.85 ± 0.29	0.86 ± 0.29	0.78 ± 0.39	<0.0001
Total protein (g/dL)	71.39 ± 4.36	71.39 ± 4.37	71.46 ± 4.16	0.7641
Smoked at least 100 cigarettes in life (%)				0.2268
Yes	39	41.25	38.12	
No	61	58.75	61.88	
Ever have 4/5 or more drinks every day (%)				0.2499
Yes	12.73	13.23	15.28	
No	87.27	86.77	84.72	
High blood pressure (%)				<0.0001
Yes	23.47	21.99	33.95	
No	76.53	78.01	66.05	
Diabetes mellitus (%)				<0.0001
Yes	7.37	5.32	14.42	
No	92.63	94.68	85.58	
Albumin (g/L)	43.23 ± 3.35	43.23 ± 3.36	41.44 ± 3.45	<0.0001
Bilirubin (umol/l)	10.43 ± 5.32	10.43 ± 5.31	9.22 ± 4.58	<0.0001
ALT (U/L)	25.75 ± 19.31	25.74 ± 19.29	30.42 ± 23.50	<0.0001
AST (U/L)	25.03 ± 17.21	25.02 ± 17.20	26.34 ± 18.25	0.1446
ALBI	−3.03 ± 0.28	−3.04 ± 0.28	−2.92 ± 0.29	<0.0001

Data were median (IQR) for skewed variables or proportions for categorical variables. BMI, body mass index; PIR, poverty income ratio; IQR, interquartile range; ALT, aminotransferase; AST, aspartate aminotransferase.

### 3.2 Association between ALBI and sarcopenia

Table 2 presents the associations between the ALBI score and sarcopenia, as demonstrated by weighted logistic regression analyses. The odds ratios (ORs) accompanied by 95% confidence intervals (CIs) indicate the likelihood of developing sarcopenia associated with increased levels of ALBI. In the unadjusted model (a one-way analysis not accounting for covariates), the OR was found to be 4.57 (95% CI: 2.63, 7.92), with a significance level of *p* < 0.001. In Model 2, which was adjusted for demographic variables, and in the fully adjusted Model 3, the ORs were 3.95 (95% CI: 2.18, 7.19) and 4.28 (95% CI: 2.29, 8.01) respectively, both demonstrating a significant association, *p* < 0.001. For further sensitivity analysis, the ALBI score was categorized into tertiles. In Model 3, participants in the highest tertile exhibited a significantly higher prevalence of sarcopenia compared to those in the lowest tertile, with an OR of 2.06 (95% CI: 1.34, 3.16), *p* < 0.005.

**Table 2:** Positive correlation between ALBI and sarcopenia.

Characteristic	Model 1 OR (95% CI)^a^, *P*-value	Model 2 OR (95% CI)^a^, *P*-value	Model 3 OR (95% CI)^a^, *P*-value
ALBI (continuous)	4.57 (2.63, 7.92) < 0.001	3.95 (2.18, 7.19) < 0.001	4.28 (2.29, 8.01) < 0.001
ALBI (categorization)			
Quartile 1	Reference	Reference	Reference
Quartile 2	1.44 (0.97, 2.15) 0.073	1.31 (0.87, 1.96) 0.2	1.31 (0.86, 1.99) 0.2
Quartile 3	2.36 (1.62, 3.43) < 0.001	2.08 (1.39, 3.10) < 0.001	2.06 (1.34, 3.16) 0.002
*P* for trend	<0.0001	<0.001	0.00154

a OR, odds ratio; CI, confidence interval.

Model 1: No covariable adjustment.

Model 2: Age, gender, race, education, marital status, and PIR adjustments.

Model 3: Age, gender, race, education, marital status, PIR, total protein, creatinine, smoking status, hypertension status, diabetes mellitus status and alcohol consumption status were all adjusted for.

### 3.3 Restricted cubic spline and threshold effect analysis

**Figure 2:**
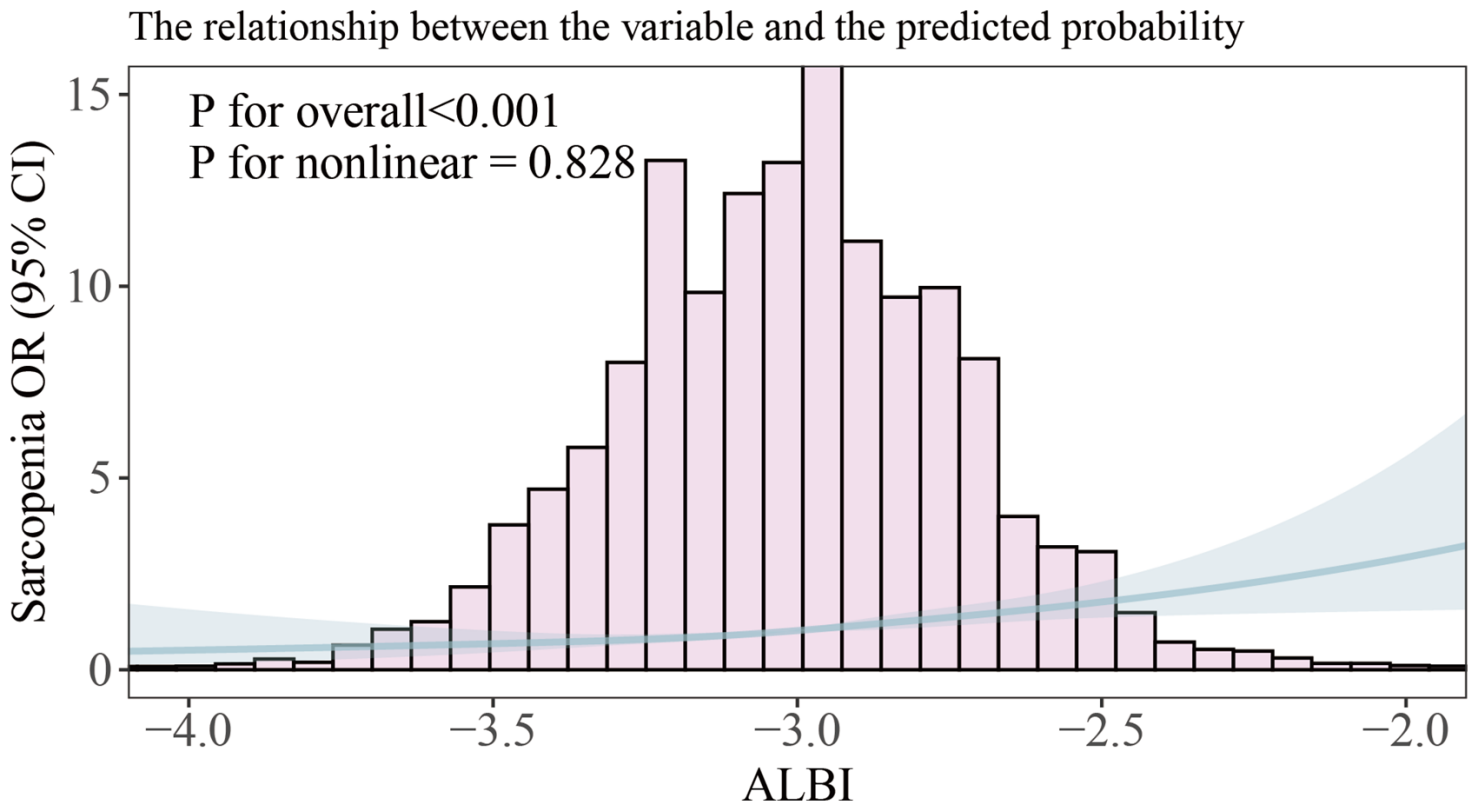
Smooth curve fitting of association between ALBI and sarcopenia. The solid portion and the shaded portion represent the predicted values and 95% confidence intervals, respectively. The model is model 3 after adjusting for all relevant covariates.

We further examined the relationship between the ALBI and sarcopenia using a restricted cubic spline (unweighted). After adjusting for relevant covariates (Model 3), a positive correlation was observed between ALBI and sarcopenia (p for overall <0.001) as illustrated in Figure 2; however, a nonlinear relationship was not established (p for nonlinear = 0.828). Additionally, our analysis included a comparison of standard linear regression with two-piecewise linear regression (unweighted), and we conducted a threshold effect analysis between the two methods using a log-likelihood ratio test (Table 3). This analysis did not identify a significant inflection point (p for likelihood ratio test = 0.399). The standard linear regression model indicated an odds ratio (OR) of 2.783 (95% CI: 1.90, 4.07), with a significance level of *p* < 0.001.

**Table 3:** Threshold effect analysis of ALBI and sarcopenia.

Sarcopenia	The effect size, (95% CI)	*P*-value
ALBI		
Model I	2.783 (1.90–4.07)	<0.001
Model II		
Inflection point	−3.283	
< −3.283	1.31 (0.264–8.52)	0.76
> − 3.283	3.039 (1.971–4.657)	<0.001
P for likelihood ratio test		0.399

Age, gender, race, education, marital status, PIR, total protein, creatinine, smoking status, hypertension status, diabetes mellitus status, and alcohol consumption status were all adjusted for.

Model I: Fitting model by standard linear regression.

Model II: Fitting model by two-piecewise linear regression.

### 3.4 Subgroup analysis

**Figure 3:**
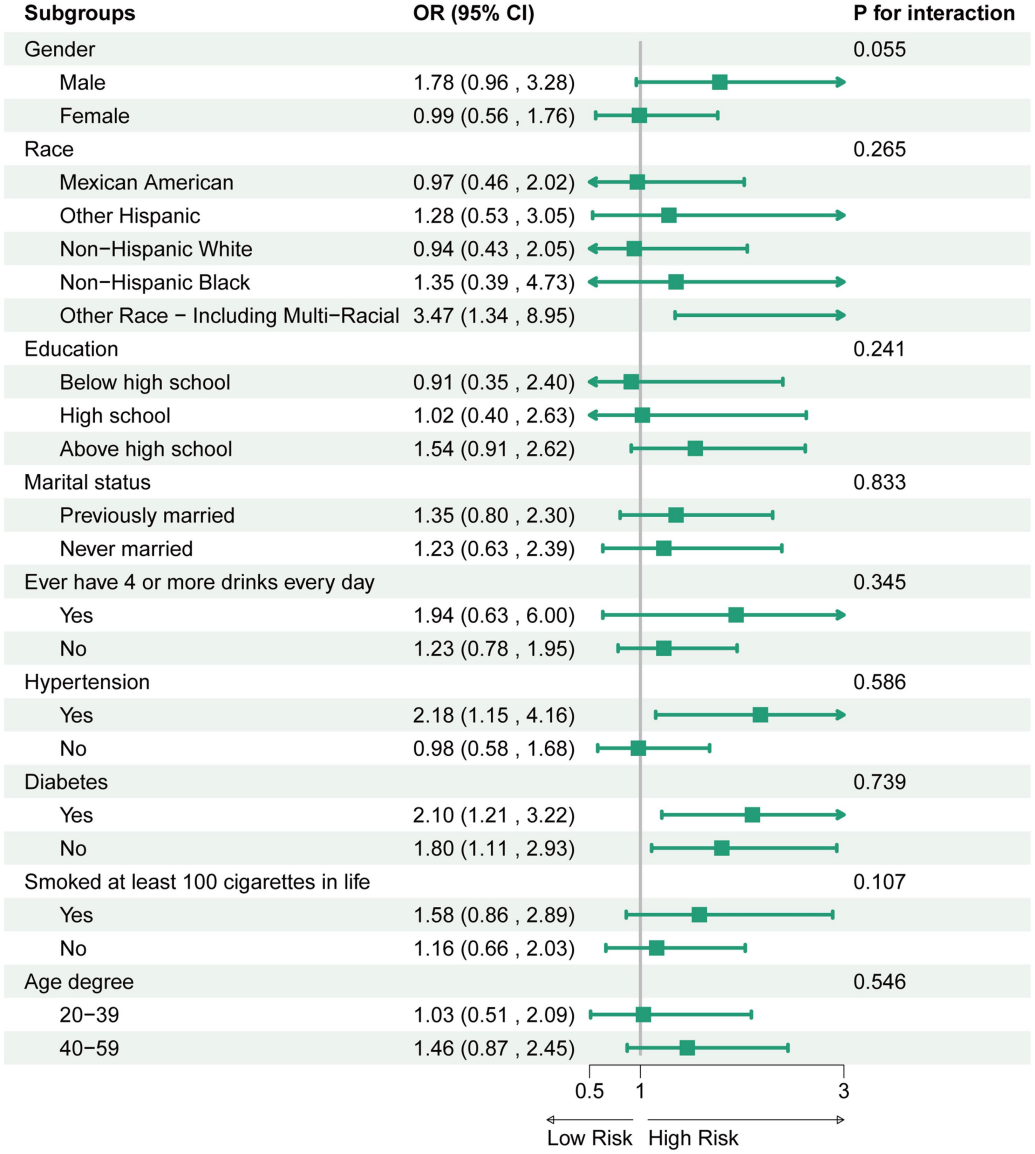
Association between ALBI and sarcopenia. For each stratification element we used model 3 (fully adjusted model) for the adjustment analysis, except for the stratification factors themselves.

To explore the possible impact of the association between ALBI and sarcopenia, we conducted stratified analyses based on gender, race, education, marital status, smoking status, alcohol consumption, as well as the hypertension and diabetes mellitus status. The findings indicated that no significant interactions were observed within these subgroups (Figure 3).

### 3.5 Comparison of the importance of variables for sarcopenia

**Figure 4:**
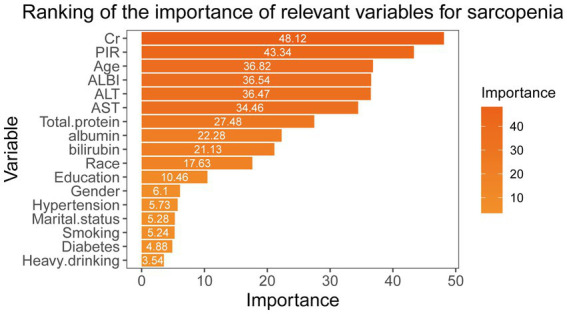
Random forest modeling to assess the importance of different variables for sarcopenia according to the Gini impurity.

We developed a random forest model to evaluate the importance of various variables linked to sarcopenia, utilizing the Gini impurity as a measure. Furthermore, we created rank diagrams to visually represent these relationships (Figure 4). Our results indicate that the albumin-bilirubin (ALBI) score is particularly significant for sarcopenia, ranking higher than conventional biomarkers such as alanine aminotransferase (ALT), aspartate aminotransferase (AST), albumin, and bilirubin.

## 4 Discussion

This study identified a positive association between increased ALBI scores and the incidence of sarcopenia among middle-aged individuals in the United States, indicating that preserving optimal liver function may notably decrease the risk of developing sarcopenia. Specifically, our analysis reveals that for each unit increase in the ALBI score, the odds of sarcopenia rise by 3.28 times in a fully adjusted model [OR (95% CI): 4.28 (2.29, 8.01), *p* < 0.001].

Sarcopenia is defined as the gradual decline in muscle mass and strength. Research suggests that the mechanisms linking liver dysfunction to muscle wasting may involve systemic inflammation, altered endocrine function, and nutrient malabsorption (28–30). For instance, chronic liver disease often leads to an inflammatory state which can promote muscle catabolism (31). Additionally, hepatic impairment can disrupt the metabolism of key nutrients essential for muscle health, further exacerbating the risk of developing sarcopenia (32). Previous research has demonstrated a notable relationship between liver function indicators and the occurrence of sarcopenia. Specifically, increased levels of liver enzymes, including alanine aminotransferase (ALT) and aspartate aminotransferase (AST), are frequently linked to a reduction in muscle mass (33). Furthermore, lower serum albumin levels, which reflect liver synthetic function, have also been implicated in the pathogenesis of sarcopenia (34).

Albumin, a protein synthesized in the liver, serves not only as a marker of nutritional status but also reflects overall health and muscle mass. Studies suggest that low serum albumin levels are associated with muscle wasting and the presence of sarcopenia. For instance, the association between hypoalbuminemia and reduced muscle mass has been documented in various populations, indicating that decreased albumin levels may serve as a prognostic marker for sarcopenia (35, 36).

Bilirubin, a byproduct of heme degradation, has recently emerged as a potential biomarker in various clinical contexts. Research indicates that higher levels of bilirubin may be inversely related to the risk of sarcopenia (37). One hypothesis is that bilirubin possesses antioxidant properties, which could potentially protect muscle cells from oxidative stress, a key factor in muscle degeneration (38). Furthermore, bilirubin levels can reflect liver health, which is closely linked to muscle metabolism. Therefore, bilirubin’s relationship with muscle mass and strength makes it a valuable component in the evaluation of sarcopenia.

The ALBI score is a novel and convenient tool to assess liver function more effectively. Unlike conventional metrics, which often focus primarily on liver enzyme levels, the ALBI score offers a more holistic view of a patient’s metabolic status, and it also suggests information related to the tumor (40–43). One significant advantage of the ALBI score is its strong association with muscle mass and nutritional status. Both albumin and bilirubin are closely related to muscle mass loss, and ALBI combines the two in a more comprehensive way. The ALBI score may serve not only as a liver function marker but also as an evaluation criteria of nutritional deficiency and muscle health (26, 44, 45). This dual utility enhances the clinical relevance of the ALBI score, making it a valuable tool in the management of patients with liver disease and those at risk of sarcopenia. Furthermore, the ALBI score is simple to calculate and can be readily obtained from routine blood tests, promoting its adoption in clinical practice. Its ability to stratify patients based on liver function and muscle health potential facilitates tailored therapeutic approaches.

In summary, the ALBI score’s comprehensive nature allows for a well-rounded evaluation of liver function, nutritional status, and muscle health, making it a promising tool for improving clinical outcomes in patients with or at risk for sarcopenia. Its ability to address both liver function and muscle mass in a single metric highlights its potential utility in clinical practice and research.

Despite the established validity of the ALBI score in contexts such as cirrhosis and hepatocellular carcinoma (46–48), its application in community-dwelling, non-cirrhotic populations warrants further consideration. One potential limitation is that the majority of existing research has predominantly focused on patients with diagnosed liver diseases, which could restrict the generalizability of the findings to middle-aged individuals who do not exhibit overt liver dysfunction. However, it is important to recognize that liver function is a critical determinant of overall health, even in the absence of diagnosed liver pathology. Subclinical liver dysfunction may still be present in asymptomatic individuals, particularly in middle-aged adults, who may exhibit metabolic syndrome or lifestyle-related risk factors that could influence liver health (49, 50).

Thus, the use of the ALBI score in our study population—a largely community-based cohort—may provide valuable insights into the subtler complexities of liver function and its implications for sarcopenia risk. By examining ALBI scores among this demographic, we aim to highlight its potential as a proactive tool for assessing liver function and muscle health long before the emergence of clinical liver disease. This approach not only broadens our understanding of liver health in a preventative context but also underscores the importance of monitoring liver function beyond traditional clinical settings. Consequently, exploring the ALBI score’s relevance in non-cirrhotic individuals can enhance its overall external validity and applicability, paving the way for early interventions that aim to mitigate sarcopenia risk in middle-aged populations.

## 5 Limitations

First, as a cross-sectional study, this research gathers data at one specific moment, research findings are dataset-specific and not causal, restricting the ability to establish causal relationships among the variables. The snapshot nature of cross-sectional research prevents the identification of longitudinal trends and potential temporal relationships, which are crucial for understanding the dynamics of the factors studied.

Second, the sample is confined to American middle-aged adults, potentially limiting the applicability of the findings to other populations. Cultural, socioeconomic, and behavioral differences among various demographic groups may influence the study variables; thus, the results may not be applicable to individuals outside this demographic.

Third, the methodology employed in diagnosing sarcopenia predominantly relied on DXA-derived muscle mass measurements, overlooking important components such as muscle strength and performance indicators (e.g., grip strength and gait speed). However, some years are missing these data in the NHANES database. These performance metrics are pivotal in the consensus definitions of sarcopenia and are essential for a comprehensive evaluation of the condition. The absence of these surrogate markers limits the robustness of the diagnosis and could lead to an underestimation of sarcopenia prevalence. This reliance on isolated measures rather than a multidimensional assessment may hinder our understanding of the complexities associated with sarcopenia and its associated health implications. Future research should strive to incorporate a more comprehensive assessment framework that encompasses both muscle mass and functional performance measures to yield more accurate insights into sarcopenia.

Lastly, some of the data collected in this study rely on self-reported measures, which can introduce subjective bias. Participants may provide socially desirable responses or may misreport their behaviors and experiences, leading to inaccuracies in the data. The dependence on subjective measures may impact the validity and reliability of the results, which should be taken into account when interpreting the findings.

In conclusion, future studies should utilize longitudinal designs to offer stronger evidence for the causal link between ALBI and sarcopenia. Furthermore, it is crucial to adopt a multidimensional approach for diagnosing sarcopenia that includes both muscle mass measurements and functional performance indicators. This comprehensive assessment will enhance the accuracy of sarcopenia diagnosis and deepen our understanding of its implications. Finally, including diverse national populations in future research will improve the generalizability of findings across different demographic groups.

## 6 Conclusion

In conclusion, our findings reveal a significant positive association between ALBI scores and the prevalence of sarcopenia among middle-aged adults in the United States. This underscores the ALBI score’s role as a comprehensive index that assesses liver function, nutritional status, and muscle health, making it a valuable tool for informing preventive strategies and clinical interventions aimed at reducing muscle loss in this high-risk demographic. Given the substantial impact of sarcopenia on quality of life and its potential to elevate the risk of adverse health outcomes, understanding the interplay between liver function and muscle integrity is crucial for enhancing clinical practices. However, further research is necessary to elucidate the mechanisms underlying this association and to determine how interventions targeting liver function might influence muscle health. Longitudinal studies and evaluations in diverse populations will be essential to validate our findings and improve their generalizability across various demographic groups.

## Data Availability

The original contributions presented in the study are included in the article/supplementary material, further inquiries can be directed to the corresponding authors.

## Footnotes

^1^ http://www.R-project.org

## References

[1] Cruz-Jentoft AJ Bahat G Bauer J Boirie Y Bruyère O Cederholm T. Sarcopenia: revised European consensus on definition and diagnosis. Age Ageing. (2019) 48:16–31. doi: 10.1093/ageing/afy169, PMID: 30312372 PMC6322506

[2] Kirk B Cawthon PM Arai H Ávila-Funes JA Barazzoni R Bhasin S. The conceptual definition of sarcopenia: Delphi consensus from the global leadership initiative in sarcopenia (GLIS). Age Ageing. (2024) 53:afae052. doi: 10.1093/ageing/afae052, PMID: 38520141 PMC10960072

[3] Chen LK Woo J Assantachai P Auyeung TW Chou MY Iijima K. Asian working group for sarcopenia: 2019 consensus update on sarcopenia diagnosis and treatment. J Am Med Dir Assoc. (2020) 21:300–307e2. doi: 10.1016/j.jamda.2019.12.012 32033882

[4] DentEMorleyJECruz-JentoftAJAraiHKritchevskySBGuralnikJ. International clinical practice guidelines for sarcopenia (ICFSR): screening, diagnosis and management. J Nutr Health Aging. (2018) 22:1148–61. doi: 10.1007/s12603-018-1139-9, PMID: 30498820

[5] Nguyen A Lee P Rodriguez EK Chahal K Freedman BR Nazarian A. Addressing the growing burden of musculoskeletal diseases in the ageing US population: challenges and innovations. Lancet Healthy Longev. (2025) 6:100707. doi: 10.1016/j.lanhl.2025.100707, PMID: 40381641

[6] Feng Z Zhao F Wang Z Tang X Xie Y Qiu L. The relationship between sarcopenia and metabolic dysfunction-associated fatty liver disease among the young and middle-aged populations. BMC Gastroenterol. (2024) 24:111. doi: 10.1186/s12876-024-03192-0, PMID: 38491346 PMC10943823

[7] Jung HN Jung CH Hwang YC. Sarcopenia in youth. Metabolism. (2023) 144:155557. doi: 10.1016/j.metabol.2023.155557, PMID: 37080353

[8] Silva TLD Mulder AP. Sarcopenia and poor muscle quality associated with severe obesity in young adults and middle-aged adults. Clin Nutr ESPEN. (2021) 45:299–305. doi: 10.1016/j.clnesp.2021.07.031, PMID: 34620332

[9] Lee C Woods PC Paluch AE Miller MS. Effects of age on human skeletal muscle: a systematic review and meta-analysis of myosin heavy chain isoform protein expression, fiber size, and distribution. Am J Physiol Cell Physiol. (2024) 327:C1400–c1415. doi: 10.1152/ajpcell.00347.2024, PMID: 39374077 PMC11684863

[10] Cruz-Jentoft AJ Sayer AA. Sarcopenia. Lancet. (2019) 393:2636–46. doi: 10.1016/S0140-6736(19)31138-9, PMID: 31171417

[11] Izzo A Massimino E Riccardi G Della Pepa G. A narrative review on sarcopenia in type 2 diabetes mellitus: prevalence and associated factors. Nutrients. (2021) 13:183. doi: 10.3390/nu13010183, PMID: 33435310 PMC7826709

[12] Feng L Gao Q Hu K Wu M Wang Z Chen F. Prevalence and risk factors of sarcopenia in patients with diabetes: a Meta-analysis. J Clin Endocrinol Metab. (2022) 107:1470–83. doi: 10.1210/clinem/dgab884, PMID: 34904651

[13] Tandon P Montano-Loza AJ Lai JC Dasarathy S Merli M. Sarcopenia and frailty in decompensated cirrhosis. J Hepatol. (2021) 75:S147–s162. doi: 10.1016/j.jhep.2021.01.025, PMID: 34039486 PMC9125684

[14] Lai JC Tandon P Bernal W Tapper EB Ekong U Dasarathy S. Malnutrition, frailty, and sarcopenia in patients with cirrhosis: 2021 practice guidance by the American Association for the Study of Liver Diseases. Hepatology. (2021) 74:1611–44. doi: 10.1002/hep.32049, PMID: 34233031 PMC9134787

[15] Sookoian S Pirola CJ. Liver enzymes, metabolomics and genome-wide association studies: from systems biology to the personalized medicine. World J Gastroenterol. (2015) 21:711–25. doi: 10.3748/wjg.v21.i3.711, PMID: 25624707 PMC4299326

[16] Testa E Malfatti F Milazzo S Cordiviola C Cotellessa T Marabotto E. Hyaluronic acid and aspartate aminotransferase levels normalized by liver function can reflect sinusoidal impairment in chronic liver disease. Liver Int. (2006) 26:439–44. doi: 10.1111/j.1478-3231.2006.01251.x, PMID: 16629647

[17] Schindhelm RK Diamant M Dekker JM Tushuizen ME Teerlink T Heine RJ. Alanine aminotransferase as a marker of non-alcoholic fatty liver disease in relation to type 2 diabetes mellitus and cardiovascular disease. Diabetes Metab Res Rev. (2006) 22:437–43. doi: 10.1002/dmrr.666, PMID: 16832839

[18] Hamoud AR Weaver L Stec DE Hinds TD Jr. Bilirubin in the liver-gut signaling Axis. Trends Endocrinol Metab. (2018) 29:140–50. doi: 10.1016/j.tem.2018.01.002, PMID: 29409713 PMC5831340

[19] Tripodi A Caldwell SH Hoffman M Trotter JF Sanyal AJ. Review article: the prothrombin time test as a measure of bleeding risk and prognosis in liver disease. Aliment Pharmacol Ther. (2007) 26:141–8. doi: 10.1111/j.1365-2036.2007.03369.x, PMID: 17593061

[20] Giannini EG Testa R Savarino V. Liver enzyme alteration: a guide for clinicians. CMAJ. (2005) 172:367–79. doi: 10.1503/cmaj.1040752, PMID: 15684121 PMC545762

[21] Deng M Ng SWY Cheung ST Chong CCN. Clinical application of albumin-bilirubin (ALBI) score: the current status. Surgeon. (2020) 18:178–86. doi: 10.1016/j.surge.2019.09.002, PMID: 31601470

[22] Ma YQ Xu XR Li J Lin Y Guan Z. Prediction of posthepatectomy liver failure in patients with hepatocellular carcinoma through ultrasound elastography. World J Gastroenterol. (2025) 31:99373. doi: 10.3748/wjg.v31.i4.99373, PMID: 39877704 PMC11718644

[23] Enkhbold C Morine Y Utsunomiya T Imura S Ikemoto T Arakawa Y. Dysfunction of liver regeneration in aged liver after partial hepatectomy. J Gastroenterol Hepatol. (2015) 30:1217–24. doi: 10.1111/jgh.12930, PMID: 25682855

[24] Kim KP Kim KM Ryoo BY Choi WM Cha WC Kang M. Prognostic efficacy of the albumin-bilirubin score and treatment outcomes in hepatocellular carcinoma: a large-scale, multi-center real-world database study. Liver Cancer. (2024) 13:610–28. doi: 10.1159/000539724, PMID: 39687041 PMC11649259

[25] European Association for Study of Liver; Asociacion Latinoamericana para el Estudio del Higado. EASL-ALEH clinical practice guidelines: non-invasive tests for evaluation of liver disease severity and prognosis. J Hepatol. (2015) 63:237–64. doi: 10.1016/j.jhep.2015.04.006 25911335

[26] Du L Xu H Fang L Qiao L Xie Y Yang C. Albumin-bilirubin score as a predictor of all-cause mortality in patients with hepatitis B virus infection: an analysis of National Health and nutrition examination survey (NHANES) 1999-2018. Prev Med Rep. (2024) 39:102639. doi: 10.1016/j.pmedr.2024.102639, PMID: 38357224 PMC10865019

[27] Zhang F Liu L Li W. Correlation of sarcopenia with progression of liver fibrosis in patients with metabolic dysfunction-associated steatotic liver disease: a study from two cohorts in China and the United States. Nutr J. (2025) 24:6. doi: 10.1186/s12937-025-01081-0, PMID: 39810142 PMC11730808

[28] Bano G Trevisan C Carraro S Solmi M Luchini C Stubbs B. Inflammation and sarcopenia: a systematic review and meta-analysis. Maturitas. (2017) 96:10–5. doi: 10.1016/j.maturitas.2016.11.006, PMID: 28041587

[29] Borba VZC Costa TL Moreira CA Boguszewski CL. Mechanisms of endocrine disease: sarcopenia in endocrine and non-endocrine disorders. Eur J Endocrinol. (2019) 180:R185–99. doi: 10.1530/EJE-18-0937, PMID: 30913536

[30] Papadopoulou SK Papadimitriou K Voulgaridou G Georgaki E Tsotidou E Zantidou O. Exercise and nutrition impact on osteoporosis and sarcopenia-the incidence of Osteosarcopenia: a narrative review. Nutrients. (2021) 13:4499. doi: 10.3390/nu13124499, PMID: 34960050 PMC8705961

[31] Allen SL Quinlan JI Dhaliwal A Armstrong MJ Elsharkawy AM Greig CA. Sarcopenia in chronic liver disease: mechanisms and countermeasures. Am J Physiol Gastrointest Liver Physiol. (2021) 320:G241–g257. doi: 10.1152/ajpgi.00373.2020, PMID: 33236953 PMC8609568

[32] Robinson S Granic A Cruz-Jentoft AJ Sayer AA. The role of nutrition in the prevention of sarcopenia. Am J Clin Nutr. (2023) 118:852–64. doi: 10.1016/j.ajcnut.2023.08.015, PMID: 37657521 PMC10636259

[33] Lian R Liu Q Jiang G Zhang X Tang H Lu J. Blood biomarkers for sarcopenia: a systematic review and meta-analysis of diagnostic test accuracy studies. Ageing Res Rev. (2024) 93:102148. doi: 10.1016/j.arr.2023.102148, PMID: 38036104

[34] Picca A Coelho-Junior HJ Calvani R Marzetti E Vetrano DL. Biomarkers shared by frailty and sarcopenia in older adults: a systematic review and meta-analysis. Ageing Res Rev. (2022) 73:101530. doi: 10.1016/j.arr.2021.101530, PMID: 34839041

[35] Amjad H Borson S. Invigorating primary care for older adults living with dementia. J Am Geriatr Soc. (2021) 69:1186–9. doi: 10.1111/jgs.17123, PMID: 33890295

[36] Ma Y Liu Y Zheng J Zheng Z Li J. Clinical significance of serum irisin, 25(OH)D3 and albumin in older adults with chronic disease and sarcopenia. Aging Clin Exp Res. (2025) 37:153. doi: 10.1007/s40520-025-03051-2, PMID: 40377817 PMC12084265

[37] Morawin B Tylutka A Bielewicz F Zembron-Lacny A. Diagnostics of inflammaging in relation to sarcopenia. Front Public Health. (2023) 11:1162385. doi: 10.3389/fpubh.2023.1162385, PMID: 37465171 PMC10351926

[38] Yamamoto S Okada H Shinagawa N Kuramoto N Ono Y Minamida M. Association between total bilirubin and sarcopenia in people with type 2 diabetes: the KAMOGAWA-A study. Endocr J. (2025). doi: 10.1507/endocrj.EJ24-0612, PMID: 40240172 PMC12340250

[39] Ge X Ciol MA Pettan-Brewer C Goh J Rabinovitch P Ladiges W. Self-motivated and stress-response performance assays in mice are age-dependent. Exp Gerontol. (2017) 91:1–4. doi: 10.1016/j.exger.2017.02.001, PMID: 28189701 PMC6118215

[40] Hiraoka A Kumada T Michitaka K Kudo M. Newly proposed ALBI grade and ALBI-T score as tools for assessment of hepatic function and prognosis in hepatocellular carcinoma patients. Liver Cancer. (2019) 8:312–25. doi: 10.1159/000494844, PMID: 31768342 PMC6873026

[41] Johnson PJ Berhane S Kagebayashi C Satomura S Teng M Reeves HL. Assessment of liver function in patients with hepatocellular carcinoma: a new evidence-based approach-the ALBI grade. J Clin Oncol. (2015) 33:550–8. doi: 10.1200/JCO.2014.57.9151, PMID: 25512453 PMC4322258

[42] Wang J Zhang Z Yan X Li M Xia J Liu Y. Albumin-bilirubin (ALBI) as an accurate and simple prognostic score for chronic hepatitis B-related liver cirrhosis. Dig Liver Dis. (2019) 51:1172–8. doi: 10.1016/j.dld.2019.01.011, PMID: 30765220

[43] Kawaguchi T Honda A Sugiyama Y Nakano D Tsutsumi T Tahara N. Association between the albumin-bilirubin (ALBI) score and severity of portopulmonary hypertension (PoPH): a data-mining analysis. Hepatol Res. (2021) 51:1207–18. doi: 10.1111/hepr.13714, PMID: 34534392

[44] Liang X Liangliang X Peng W Tao Y Jinfu Z Ming Z. Combined prognostic nutritional index and albumin-bilirubin grade to predict the postoperative prognosis of HBV-associated hepatocellular carcinoma patients. Sci Rep. (2021) 11:14624. doi: 10.1038/s41598-021-94035-5, PMID: 34272447 PMC8285529

[45] Kaibori M Hiraoka A Matsui K Matsushima H Kosaka H Yamamoto H. Predicting complications following surgical resection of hepatocellular carcinoma using newly developed neo-Glasgow prognostic score with ALBI grade: comparison of open and laparoscopic surgery cases. Cancers (Basel). (2022) 14:1402. doi: 10.3390/cancers14061402, PMID: 35326554 PMC8946274

[46] Elsabaawy M Badran H Ragab A Abdelhafiz R Nageeb M Ashour R. ALBI-sarcopenia score as a predictor of treatment outcomes in hepatocellular carcinoma. Sci Rep. (2025) 15:14621. doi: 10.1038/s41598-025-97295-7, PMID: 40287454 PMC12033259

[47] Guan MC Ding Q Zhao Q Li N Zhang RX Zhang SY. Albumin-bilirubin grade as an alternative to child-Pugh class for evaluating liver function within staging systems for hepatocellular carcinoma. Discov Oncol. (2025) 16:394. doi: 10.1007/s12672-025-02187-x, PMID: 40133693 PMC11936848

[48] Nishikawa H Enomoto H Yoh K Iwata Y Sakai Y Kishino K. Combined albumin-bilirubin grade and skeletal muscle mass as a predictor in liver cirrhosis. J Clin Med. (2019) 8:782. doi: 10.3390/jcm8060782, PMID: 31159435 PMC6617543

[49] Wang JJ Zheng Z Zhang Y. Association of overweight/obesity and overweight/obesity-related metabolic dysfunction-associated steatotic liver disease in young adults with coronary artery calcification later in life. Diabetes Obes Metab. (2024) 26:3860–7. doi: 10.1111/dom.15733, PMID: 38934214

[50] Durak H Çetin M Emlek N Özyıldız AG Ergül E Duman H. Association of subclinical carotid artery atherosclerosis assessed by carotid ultrasound with nonalcoholic fatty liver disease fibrosis score in young and middle-aged men. Int J Cardiovasc Imaging. (2024) 40:1979–86. doi: 10.1007/s10554-024-03193-w, PMID: 39012403

